# The Effect of Repetitive Transcranial Magnetic Stimulation on Gamma Oscillatory Activity in Schizophrenia

**DOI:** 10.1371/journal.pone.0022627

**Published:** 2011-07-27

**Authors:** Mera S. Barr, Faranak Farzan, Tamara Arenovich, Robert Chen, Paul B. Fitzgerald, Zafiris J. Daskalakis

**Affiliations:** 1 Schizophrenia Program, Department of Psychiatry, Centre for Addiction and Mental Health, University of Toronto, Toronto, Ontario, Canada; 2 Biostatistical Consultant, Centre for Addiction and Mental Health, Toronto, Ontario, Canada; 3 Division of Neurology, Toronto Research Institute, University of Toronto, Toronto, Ontario, Canada; 4 Monash Alfred Psychiatry Research Centre, The Alfred and Monash University School of Psychology and Psychiatry, Melbourne, Victoria, Australia; International School for Advanced Studies, Italy

## Abstract

**Background:**

Gamma (γ) oscillations (30–50 Hz) have been shown to be excessive in patients with schizophrenia (SCZ) during working memory (WM). WM is a cognitive process that involves the online maintenance and manipulation of information that is mediated largely by the dorsolateral prefrontal cortex (DLPFC). Repetitive transcranial magnetic stimulation (rTMS) represents a non-invasive method to stimulate the cortex that has been shown to enhance cognition and γ oscillatory activity during WM.

**Methodology and Principal Findings:**

We examined the effect of 20 Hz rTMS over the DLPFC on γ oscillatory activity elicited during the N-back task in 24 patients with SCZ compared to 22 healthy subjects. Prior to rTMS, patients with SCZ elicited excessive γ oscillatory activity compared to healthy subjects across WM load. Active rTMS resulted in the reduction of frontal γ oscillatory activity in patients with SCZ, while potentiating activity in healthy subjects in the 3-back, the most difficult condition. Further, these effects on γ oscillatory activity were found to be specific to the frontal brain region and were absent in the parieto-occipital brain region.

**Conclusions and Significance:**

We suggest that this opposing effect of rTMS on γ oscillatory activity in patients with SCZ versus healthy subjects may be related to homeostatic plasticity leading to differential effects of rTMS on γ oscillatory activity depending on baseline differences. These findings provide important insights into the neurophysiological mechanisms underlying WM deficits in SCZ and demonstrated that rTMS can modulate γ oscillatory activity that may be a possible avenue for cognitive potentiation in this disorder.

## Introduction

Gamma (γ) oscillations (30–50 Hz) are associated with working memory (WM). WM involves the maintenance and manipulation of information [1] and has been shown to increase γ oscillations with increases in WM load in healthy subjects [2], particularly in the dorsolateral prefrontal cortex (DLPFC; [3]). Schizophrenia (SCZ) patients have marked deficits in WM [4] that has been attributed to altered γ oscillatory activity. For example, we demonstrated that SCZ patients compared to healthy subjects elicit excessive γ oscillatory activity while performing the N-back task at all WM loads that was accompanied by impaired performance [5]. Although previous studies provide evidence for reduced γ oscillatory activity in SCZ patients during cognitive control [6] and sensory oddball [7] tasks, recent findings suggest that γ oscillatory activity is excessive in the frontal cortex during WM performance [2], [5]. Excessive γ oscillatory activity has also been shown in the posterior cortex in patients with SCZ during visual stimulation [3], [8]. Altered γ oscillatory activity in SCZ patients may therefore be related to impaired WM function in this disorder.

Animal studies have shown that γ oscillations during WM are supported by γ –aminobutyric acid (GABA) interneurons in the DLPFC [9], [10], [11]. Specifically, GABAergic activity may be involved in the generation and inhibition of γ oscillations [12], [13], [14], [15], a mechanism that has been shown to be impaired in SCZ [16], [17], [18], [19]. In line with these studies, Farzan et al. (2010) measured neurophysiological indices of GABA_B_ receptor inhibition from the DLPFC in SCZ patients compared to bipolar disordered patients and healthy subjects through combined transcranial magnetic stimulation (TMS) electroencephalography (EEG) [20]. It was demonstrated that the inhibition of γ oscillations was significantly reduced in DLPFC of SCZ patients compared to the other 2 groups [20]. It is possible, therefore, that deficits in the inhibition of γ oscillations in the DLPFC in SCZ results in excessive γ activity as was previously reported [5], which may contribute to WM deficits in this disorder. By contrast, in healthy subjects it was demonstrated that repetitive TMS (rTMS) over the DLPFC selectively enhanced γ oscillatory activity that was most pronounced in 3-back [3], which may be related to its ability to potentiating effects on GABAergic neurotransmission [21], [22].

## Methods

### Objective

The objective of this study was to administer high frequency rTMS over the DLPFC to measure its effect on γ oscillatory activity during the N-back task in patients with SCZ and healthy subjects. It was hypothesized that excessive γ oscillatory activity in patients with SCZ would be reduced with rTMS compared to sham stimulation and in contrast to healthy subjects.

### Participants

Twenty-four (males = 14; females = 10) patients with a diagnosis of SCZ or schizoaffective disorder confirmed by the Structured Clinical Interview for DSM-IV [23] and 22 (males = 11; females = 11) healthy individuals participated in this study. Eighteen of the twenty-two subjects overlapped with a study previously published [3]. All subjects were right handed confirmed using the Oldfield Handedness Inventory [24]. Patients with SCZ were all treated with antipsychotic medication (14.4±10.9 mg olanzapine, 6 patients; 233.3±230.9 mg clozapine, 3 patients; 5.2±3.0 mg risperidone, 7 patients; 733.3±416.3 mg of quetiapine, 3 patients; 2.4±1.6 mg fluphenazine, 3 patients ; 25 mg haloperidol, 1 patient; 15 mg aripiprazole, 1 patient). Demographic data of the subject groups are shown in Table 1. The subject groups were similar in age (t_(44)_ = −0.754, p = 0.455), but differed in education (independent t-tests: t_(44)_ = 2.954, *p*<0.05; Table 1). Severity of psychopathology was evaluated using the positive and negative symptom scale (PANSS; [25]), scale for the assessment of negative symptoms (SANS; [26]) and the Calgary Depression Scale (CDS; [27]; Table 1). Exclusion criteria for all subjects included a history of substance abuse or dependence in the last 6 months determined through the DSM-IV, a concomitant major and unstable medical or neurologic illness or pregnant. In healthy subjects the presence of psychopathology was ruled out through the personality assessment screener (PAS; Psychological Assessment Resources, Inc).

**Table 1:** Demographic Data for Healthy Subjects (HS) and patients with schizophrenia (SCZ) and the assessment of psychotic symptoms in patients with SCZ rTMS (±) 1 standard deviation.

		HS	SCZ
Age		44.500 (±) 11.43	47.21 (±) 12.80
Age Range		23–61	23–70
Female (n)		11	10
Male (n)		11	14
PANSS Scores	Positive	NA	17.50 (±) 6.40
	Negative	NA	14.71 (±) 7.09
	Global	NA	27.45 (±) 7.60
	Total	NA	57.54 (±) 16.05
Psyrats Score	Total	NA	13.42 (±) 13.52
CDS Score	Total	NA	2.94 (±) 2.99
SANS Score	Total	NA	37.67 (±) 21.42

### Description of Procedures

This study was a randomized, double-blind, placebo-controlled design. Patients with SCZ and healthy subjects were randomized into two groups allocated to receive either active or sham rTMS and were blind to their group assignment. Furthermore, active and sham rTMS was administered by treatment nurses who were not involved in any other the experimental measures or data analysis. The clinical rater and the experimenter who analyzed and interpreted the data were both blind to the rTMS group assignment. The experiment took place over two testing days. On the first day, subjects performed the N-back test while their EEG was recorded. One week later, rTMS was administered over the DLPFC followed by the final testing of the N-back task. The final N-back task was performed approximately 20 minutes following the rTMS administration to allow for cortical plasticity to take place as well as for the placement of the EEG cap. These two N-back testing sessions will be referred to ‘pre’ and ‘post’ measures relative to the rTMS administration here on in.

#### N-Back Task

**Figure 1:**
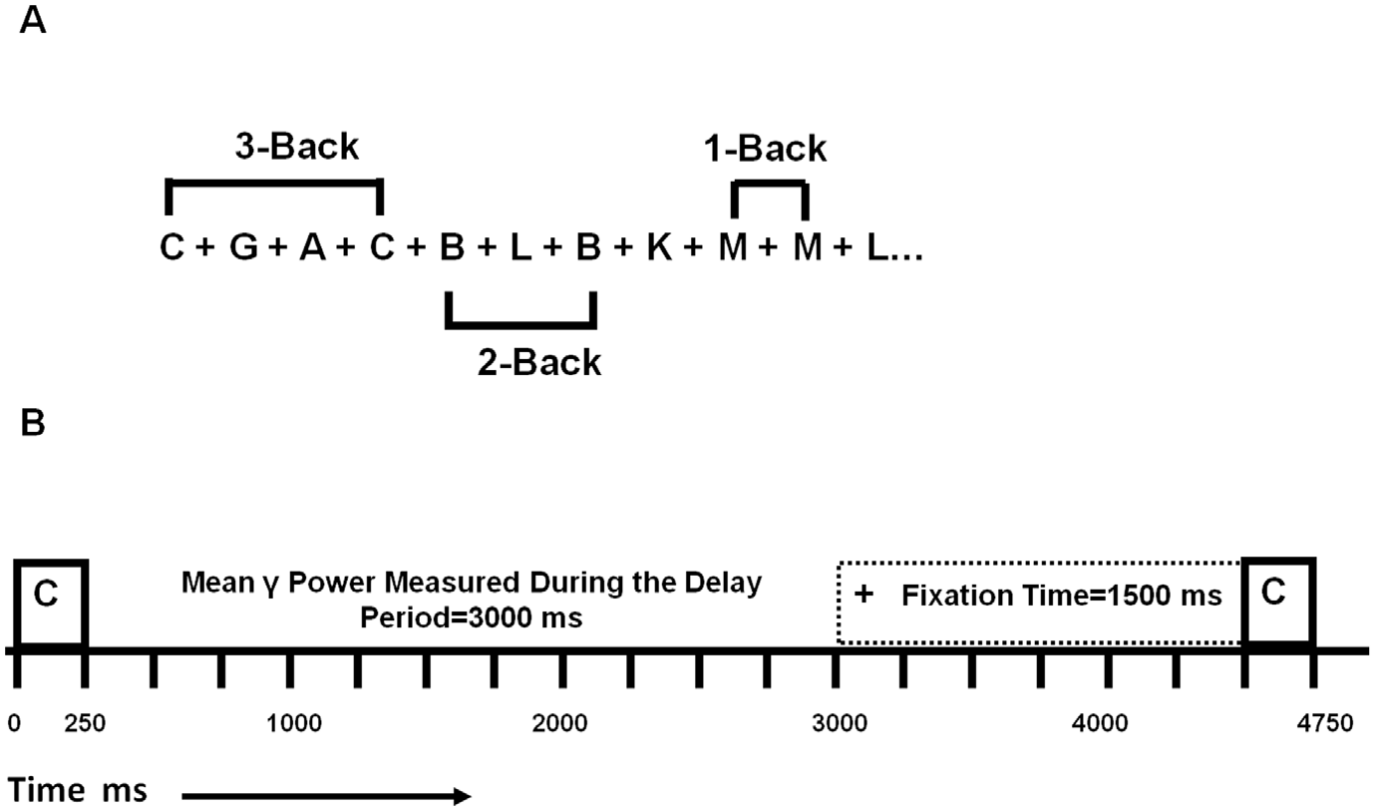
A representation of the 1-, 2- and 3-back conditions that were completed in a randomized order by patients with schizophrenia (SCZ) and healthy subjects (HS) pre-post rTMS. Subjects were required to push one button (target) if the current letter was identical to the letter presented “N” trials back; otherwise the participants pushed a different button (non-target). Correct responses for target (TC) and non-target (NTC) were included in the data analysis (A). The timing of one trial from the presentation of a one letter separated by a (+) sign followed by a subsequent letter for a total time of 3000 msec (B).

Subjects performed the N-back task while their EEG was recorded (STIM2, Neuroscan, U.S.A.) pre and post rTMS. Stimuli were presented on a computer monitor one at a time and participants were required to push one button (target) if the present stimulus was identical to the stimulus presented “N” trials back; otherwise, subjects pushed a different button (non-target). Thus, the effect of increasing cognitive demand on oscillatory activity was tested by varying the “N” in the 1-, 2- and 3-back conditions. Stimuli consisted of black capital letters presented for 250 msec followed by a delay period of 3000 msec during which the subject was required to respond (Figure 1). In the 1- and 2-back conditions, stimuli were presented continuously for 15 minutes and for 30 minutes in the 3-back condition. The 3-back was administered for double the length of time to ensure a satisfactory number of correct responses were contained for the data analysis (Table 2). The number of target letters in each condition was: 46 of 198 (23.2%) 1-back; 31 of 197 trials (15.7%) 2-back, and 59 of 400 trials (14.6%) 3-back condition. The N-back task took 1 hour for subjects to complete with the order of conditions randomized and counterbalanced to control for order effects.

**Table 2:** Total number (TC+NTC) of trials analyzed for healthy subjects (HS) and patients with schizophrenia (SCZ) in the 1-, 2-, and 3-back task conditions pre- post-rTMS (±) 1 standard deviation.

No of Trials	Condition	HS				SCZ			
		Pre		Post		Pre		Post	
		Active	Sham	Active	Sham	Active	Sham	Active	Sham
	**1-Back**	130.91	130.00	127.09	114.81	82.00	84.17	104.00	81.00
**(±) SD**		39.77	24.61	42.37	48.82	42.21	43.79	35.60	41.97
	**2-Back**	150.00	130.36	139.82	104.91	101.92	96.08	54.42	90.50
**(±) SD**		32.75	33.14	43.21	26.36	42.39	44.07	36.39	45.57
	**3-Back**	283.82	225.64	264.45	181.64	157.27	168.42	122.08	131.83
**(±) SD**		76.22	62.92	79.65	77.80	73.38	76.71	59.42	78.26

#### Repetitive TMS

Repetitive TMS was administered using a Medtronic MagPro stimulator (Medtronic, Inc., U.S.A.) with a 70 mm diameter figure-of-8 coil to the right and left DLPFC at 20 Hz, 90% resting motor threshold for 25 trains comprising of 30 pulses per train, inter-train interval of 30 seconds for a total of 750 pulses per hemisphere in accordance with published safety guidelines [28]. The total time for the rTMS administration was 25 minutes, 12.5 minutes per hemisphere. The resting motor threshold was defined as the lowest intensity that produced a motor evoked potential of at least 50 µV in 50% of the trials delivered. Sham stimulation was delivered at the same rTMS parameters as active stimulation with the coil held in a single wing-tilt position at 90 degrees to induce similar somatic sensations as in the active stimulation with minimal direct brain effects. The order of stimulation (right then left versus left then right) was also randomized and counterbalanced to control for order effects.

#### DLPFC Site Localization

**Figure 2:**
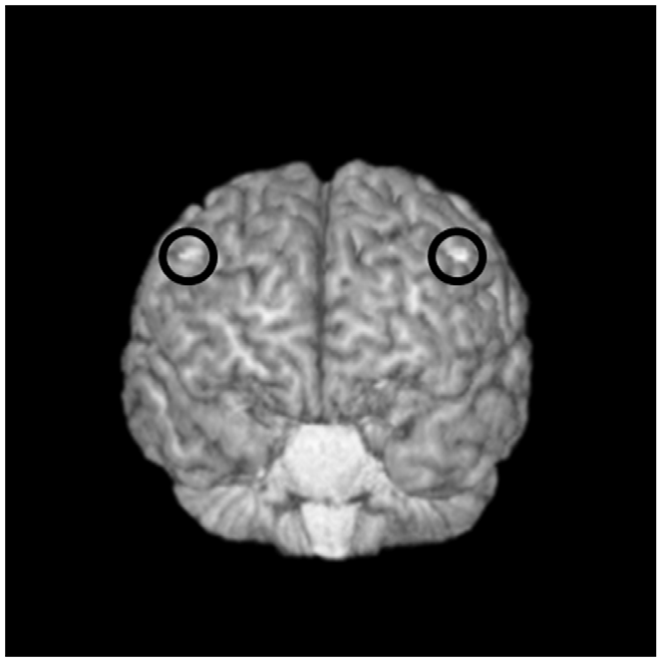
Targeting the Dorsolateral Prefrontal Cortex (DLPFC) for rTMS stimulation. Transverse view from a single subject with exposed cortex and overlap of Brodmann areas 9 & 46, highlighted (white) on a T1-weighted 3D MRI. Using MRI-to-MiniBird co-registration, the centre of the TMS coil was held over this region.

The localization of the DLPFC was determined through neuronavigational techniques using the MINIBIRD system (Ascension Technologies) combined with MRIcro/reg software using a T1-weighted MRI scan obtained for each subject with seven fiducial markers in place. Repetitive TMS was targeted at the junction of the middle and anterior one-third of the middle frontal gyrus (Talairach coordinates (x, y, z) = +/−50, 30, 36) corresponding with posterior regions of Brodmann area 9 (BA9), and overlapping with the superior region of BA46 (Figure 2). The selection of this site was based on recent meta-analyses of functional imaging studies that examined WM and the activation of the DLPFC [29], [30], [31].

#### EEG Measurement of Evoked γ Oscillatory Activity

Evoked and induced oscillatory methods have both been used to examine oscillatory activity elicited during WM. These two analytical methods differ in their relationship with the stimulus onset. Evoked oscillatory responses are phase-locked to the stimulus onset with a fixed latency following stimulus onset and are measured by stimulus-triggered averaging of responses in a time domain [32]. By contrast, induced oscillatory activity is not phase-locked to stimulus onset and appears as a jitter in latency that varies from trial to trial, thus, these responses are cancelled out when trials are averaged [32]. In other words, evoked responses are characterized by a constant time and phase relationship with the stimulus while a loose temporal relationship with the stimulus characterizes induced activity. In the frontal region, evoked and induced oscillatory activities have been shown to overlap during the N-back task [33]. Furthermore, it was initially suggested that early evoked activities reflect perceptual processes while induced activities reflect more attentional and WM processes [32]. However, there is increasing evidence for the modulation of evoked oscillatory activity with WM load particularly when longer delay periods are examined [2], [3], [34] thereby suggesting that evoked oscillations are involved in both attentional and other WM processes (e.g., retention and retrieval) and are not simply limited to perception. In addition, during EEG recordings both eye movement and cranial musculature artefact have been a concern with the measurement of oscillatory activity during cognitive paradigms specifically in the γ band. For example, Yuvel-Greenberg et al (2008) measured microsaccadic eye movement and EEG simultaneously while subjects performed a cognitive task and demonstrated that induced γ oscillatory activity corresponded with microsaccadic eye movement rather than neuronal processing [35]. It was concluded that evoked measurement of γ oscillatory activity is less susceptible to eye movement artefact owing to the fact that this activity is cancelled out when multiple trials are averaged. This also applies to activity from cranial musculature which is another source of artefact that has been a concern with the examination of γ oscillatory activity during cognition [36]. That is, the modulation of γ band activity has been associated with cranial musculature as the difficulty of cognitive tasks increases. However, it has been reported that such artefact due to cranial musculature is characterized by irregular spikes present across spectral frequencies [37] and therefore are more likely to influence induced measurement of γ oscillatory activity. The measurement of evoked oscillatory activity during WM performance is less susceptible to both eye movement and cranial musculature artefact [38]. As such, we measured mean evoked γ power from frontal electrodes while subjects completed the N-back task before (pre) and after (post) rTMS was administered over the right and left DLPFC.

#### EEG Recording

EEG data were acquired using a 64-electrode cap and Synamps2 DC-coupled EEG system (Compumedics, U.S.A.). Four electrodes placed on the outer side of each eye, above, and below the left eye were used to monitor eye movement artefact. Data was recorded at a rate of 1000 Hz DC and with a 0.3 to 200 Hz band pass hardware filter. Electrode impedances were lowered to <5 kΩ. All channels were referenced to an electrode placed posterior to the Cz electrode.

#### Offline EEG processing

We measured mean evoked oscillatory power over the delay period according to published protocol [3]. Data was filtered off-line using a 1 to 100 Hz band pass zero phase shift filter (slope, 24 dB/oct). Epochs were defined as −1000 to +3095 msec relative to the cue onset and were baseline corrected with respect to the prestimulus interval (−1000 to cue onset). All trials were manually inspected and any error trials or epochs containing artefact (movement or electrooculogram exceeding +/−50 µV) were excluded from further analysis.

### Ethics

All subjects provided their written informed consent and the protocol was approved by the Centre for Addiction and Mental Health in accordance with the declaration of Helsinki.

### Data Analysis and Statistical Methods

#### Behavioural Analysis

The total number of correct trials (target correct (TC) and non-target correct (NTC)) including those trials rejected due to artefact were included in the data analysis for WM performance and reaction time. Two separate repeated measures ANOVA were conducted on the baseline measures of WM performance and reaction time with N-back as a within-subject factor (1- versus 2-versus 3-back) and group (patients with SCZ versus healthy subjects) as the between-subject factor. Two separate mixed model repeated measures (MMRM) for WM and reaction were then performed on change score (post rTMS-pre rTMS) with Group (patients with SCZ versus healthy subjects) and rTMS (active versus sham) as between-subject factors and WM load (1- versus 2- versus 3-back) as the within-subject factor with a significance level set at p<0.05. Interaction effects were further examined with Bonferroni-adjusted pairwise comparisons (SAS System v.9.1.3; SAS Institute, NC, USA).

#### EEG Analysis

Artefact-free EEG data were imported into MATLAB (The MathWorks, Inc. Natick, MA, USA) using the EEGLAB toolbox [39] for subsequent analysis. Evoked oscillatory power for each frequency band was examined using a zero phase shift Hamming based FIR filter (in the order of 100) to decompose the EEG signal into δ (1–3.5 Hz); θ (4–7 Hz); α (9–12 Hz); β (14–28 Hz) and γ, (30–50 Hz) and averaged over the delay period (0–3000 msec from cue onset) for the target correct (TC) and non-target correct (NTC) responses for each WM load pre and post rTMS for each subject. Mean evoked oscillatory power was then assessed during these responses (TC and NTC) from the frontal electrodes (AF3, AF4, F5, F3, F1, FZ, F2, F4, and F6), and averaged for each subject. Since spectral analysis of EEG activity is often not normally distributed [40], the data was log transformed prior to analysis. A series of five (across oscillatory power in the 5 frequency bands) repeated measures ANOVA were conducted at baseline with N-back condition (1- versus 2- versus 3-back) as a within subject factor and group (patients with SCZ versus healthy subjects) as the between subject factor with a significance level set at p<0.05. Next, a series of five MMRM were performed on change score (post rTMS-pre rTMS) with Group (patients with SCZ versus healthy subjects) and rTMS (active versus sham) as between-subject factors and WM load (1- versus 2- versus 3-back) as the within-subject factor with a significance level set at p<0.05. The exclusion of the time (pre rTMS versus post rTMS) within subject factor was chosen to simplify the model to allow for an easier interpretation of a 3-way interaction versus a 4-way interaction term that would have been highly unstable. As such, the MMRM analyses were carried out on change scores for oscillatory power and behavioural data (post rTMS-pre rTMS). Interaction effects were further examined with Bonferroni-adjusted pairwise comparisons (SAS System v.9.1.3; SAS Institute, NC, USA). MMRM analyses were therefore conducted on the change in WM performance and oscillatory activity following rTMS as the 4-way interaction (e.g., group (patients with SCZ versus healthy subjects), rTMS (active versus sham) and N-back (1- versus 2- versus 3-back)) would have been unstable as directed by our biostatistician. Subsequent exploratory analyses that were not set out as a priori hypotheses were carried out using repeated measures ANOVAs.

## Results

### Baseline N-Back Performance

A repeated measures ANOVA on N-back performance pre rTMS found a significant main effect of N-back (F_(2,78)_ = 52.29; p = 0.0001) such that performance decreased with increased WM load across subjects. A significant group effect was also revealed (F_(1,39)_ = 6.87; p = 0.012) reflecting poorer N-back performance in patients with SCZ compared to healthy subjects. The interaction was not significant. A repeated measures ANOVA on response time pre rTMS also revealed a significant main effect of N-back (F_(2,84)_ = 29.45; p = 0.0001) indicative of increased response time with increased WM load. The group and interaction were not significant.

### Baseline Evoked γ Power

Prior to rTMS, a repeated measures ANOVA on evoked γ power revealed a significant main effect of N-back (F_(2,80)_ = 4.21; p = 0.018) reflecting the increase in γ power from 1- to 2-back (t = −2.61, df = 43, p = 0.012) with a subsequent decrease in γ power from the 2- to 3-back (t = 2.37, df = 42, p = 0.022) found through paired t-tests. The N-back×group interaction was also found significant (F_(2,80)_ = 10.38; p = 0.018) whereby an independent t-test revealed reduced γ power in the 3-back condition (t = −4.68, df = 1,40, p = 0.004). To examine if this effect was due to a difference in performance, first an independent t-test was conducted on 1-back performance of SCZ patients compared to healthy subjects' 3-back performance and found no difference (t = −1.23, df = 42, p = 0.226) followed by an independent t-test on γ power which found that patients still generated significantly greater activity (t = −3.70, df = 43, p = 0.001) at equivalent performance levels. Finally, the group effect was significant (F_(1,40)_ = 9.15; p = 0.004) reflective of significantly greater γ power generated by the SCZ group compared to the healthy subject group.

### EEG Spectral Analysis of Other Frequency Bands

Four separate repeated measures ANOVA conducted on the δ, θ, α, and β frequency bands revealed a group effect in β power (F_(1,38)_ = 11.68; p = 0.002) such that SCZ patients generated significantly reduced β activity compared to healthy subjects.

### Change in N-Back Behavioural Performance

N-back performance accuracy was significantly worse in patients with SCZ compared to healthy subjects pre and post rTMS; however, there were no significant improvements in N-back performance accuracy following either active or sham rTMS stimulation in either subject group found through the MMRM analysis (Table 3). Similarly, the MMRM analysis found no effect of rTMS on response time in either subject group (Table 3).

**Table 3:** Working memory (WM) behavioural performance (%) and reaction time (RT; msec) during the N-back in healthy subjects (HS) versus patients with schizophrenia (SCZ) pre-post either active or sham rTMS (±) 1 standard deviation pre-post rTMS.

Behavioural	Condition	HS				SCZ			
		Pre		Post		Pre		Post	
		Active	Sham	Active	Sham	Active	Sham	Active	Sham
**Score (%)**	**1-Back**	83.19	92.12	83.58	94.01	79.05	77.33	76.22	69.42
**(±) SD**		11.61	2.26	7.80	3.15	15.16	18.31	21.07	29.75
	**2-Back**	74.18	88.03	72.49	89.93	66.25	72.46	62.95	79.61
**(±) SD**		16.29	13.57	13.32	6.87	11.58	22.99	24.11	16.72
	**3-Back**	66.86	77.41	60.85	62.65	48.38	59.99	48.55	57.46
**(±) SD**		11.58	15.39	3.12	3.71	16.08	20.84	15.40	15.97
**RT (msec)**	**1-Back**	793.56	720.78	756.73	706.81	945.02	754.36	874.35	796.48
**(±) SD**		147.18	99.26	90.68	134.15	276.11	192.41	277.71	192.70
	**2-Back**	924.42	891.49	861.96	861.11	1130.73	865.01	1012.17	902.22
**(±) SD**		257.44	272.74	204.21	216.49	434.34	245.49	340.66	288.48
	**3-Back**	990.42	970.08	956.42	865.53	1114.51	938.66	937.35	929.60
**(±) SD**		317.13	247.70	257.75	203.11	277.71	236.39	322.09	252.02

### Change in Evoked γ Power

**Figure 3:**
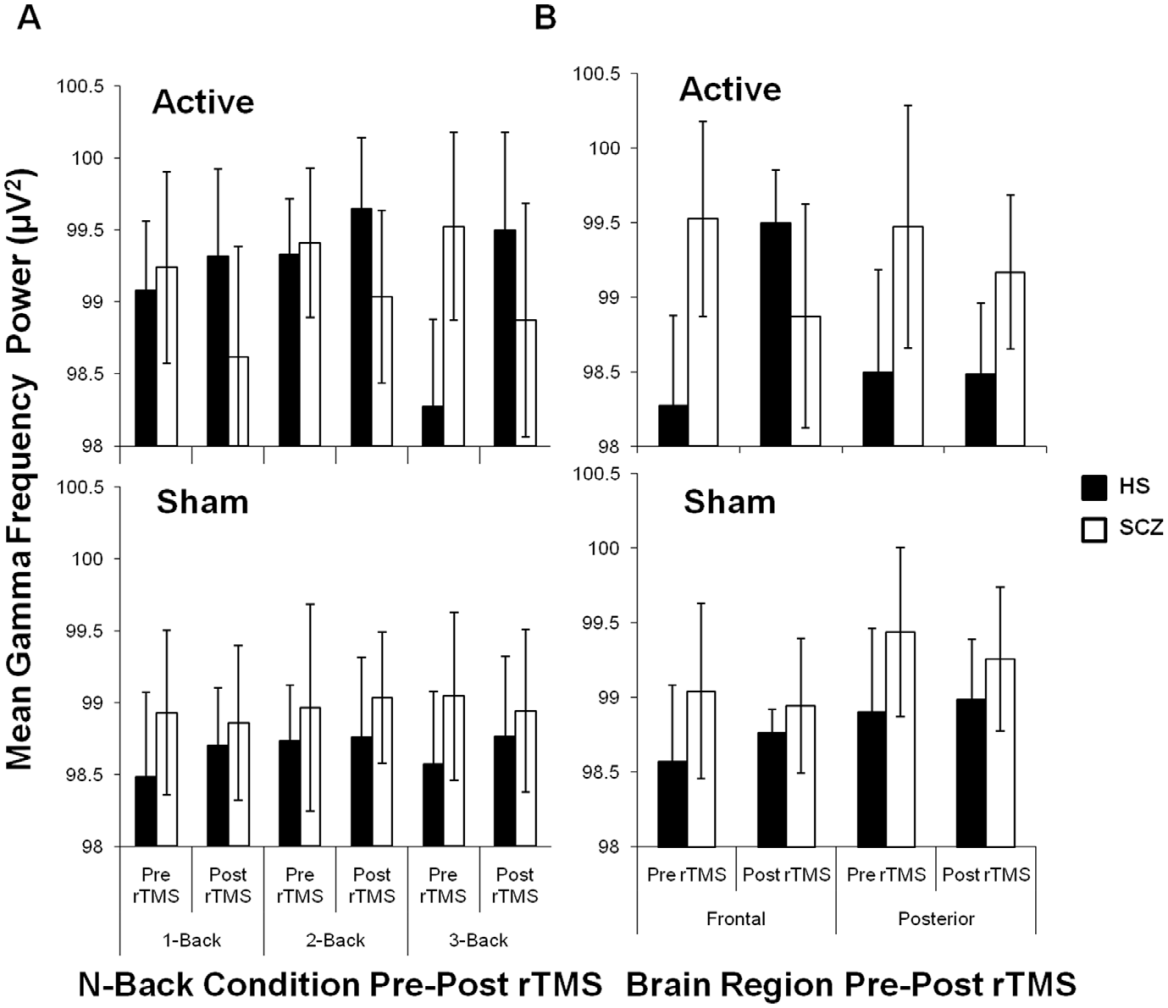
Mean log transformed gamma oscillatory power (γ; 30–50 Hz; uV^2^) for target correct (TC) and non-target correct (NTC) responses during the N-back task pre-post rTMS in healthy subjects (HS; N = 22) versus patients with schizophrenia (SCZ; N = 24) (A). Mean log transformed γ oscillatory power (uV^2^) for target correct (TC) and non-target correct (NTC) responses during the 3-back condition measured from the frontal and parieto-occipitalbrain regions pre-post rTMS in patients with schizophrenia (SCZ; N = 24) and healthy subjects (HS; N = 22) (B). Bars represent (±) 1 standard deviation.

The MMRM analysis on the change in mean γ power (post rTMS γ power-pre rTMS γ power) found a significant Group difference between patients with SCZ and healthy subjects (F_(1,42)_ = 18.23; p = 0.0001). Further, significant Group×rTMS (F_(1,42)_ = 10.37; p = 0.0025) and Group×N-back condition (F_(2,42)_ = 6.41; p = 0.0037) interaction effects were found. The Group×rTMS×N-back interaction was also significant (F_(2,42)_ = 3.75; p = 0.0317; Figure 3A) indicating that the effects of Group and rTMS differed across WM load. A series of 15 Bonferroni-adjusted pairwise comparisons were then performed to better understand this 3-way interaction. Active stimulation was found to reduce γ power in patients with SCZ, while potentiating γ power in healthy subjects. Moreover, this effect of active stimulation on γ power differed significantly in patients with SCZ compared to healthy subjects in the 3-back condition (p<0.0001), while trending differences were observed in the 1- (p = 0.0750) and 2-back (p = 0.0795) conditions. To explore whether the effects of rTMS in the 3-back condition were specific to the frontal brain region, we compared mean γ power from the frontal versus parieto-occipital brain region (electrodes: OZ, O1, O2, PO3, and PO4). A repeated measures ANOVA was conducted with Group, Time, and rTMS as between subject factors and brain region as a within subject factor (SPSS 15.0, SPSS Inc. Chicago, Illinois, USA) and a significant Time×Region×Subject×rTMS (F_(1, 35)_ = 9.072; p = 0.005; Figure 3B) interaction was revealed. Pairwise comparisons found no differences in the parieto-occipital brain region in both subject groups following both active and sham stimulation. The effects of active rTMS on γ oscillatory activity therefore were specific to the frontal brain region in the 3-back condition. These results suggest that active rTMS over the DLPFC reduced frontal γ power in patients with SCZ, while potentiating this activity in healthy subjects that was most pronounced in the 3-back condition with the greatest difficulty.

### Change in EEG Spectral Analysis of Other Frequency Bands

**Figure 4:**
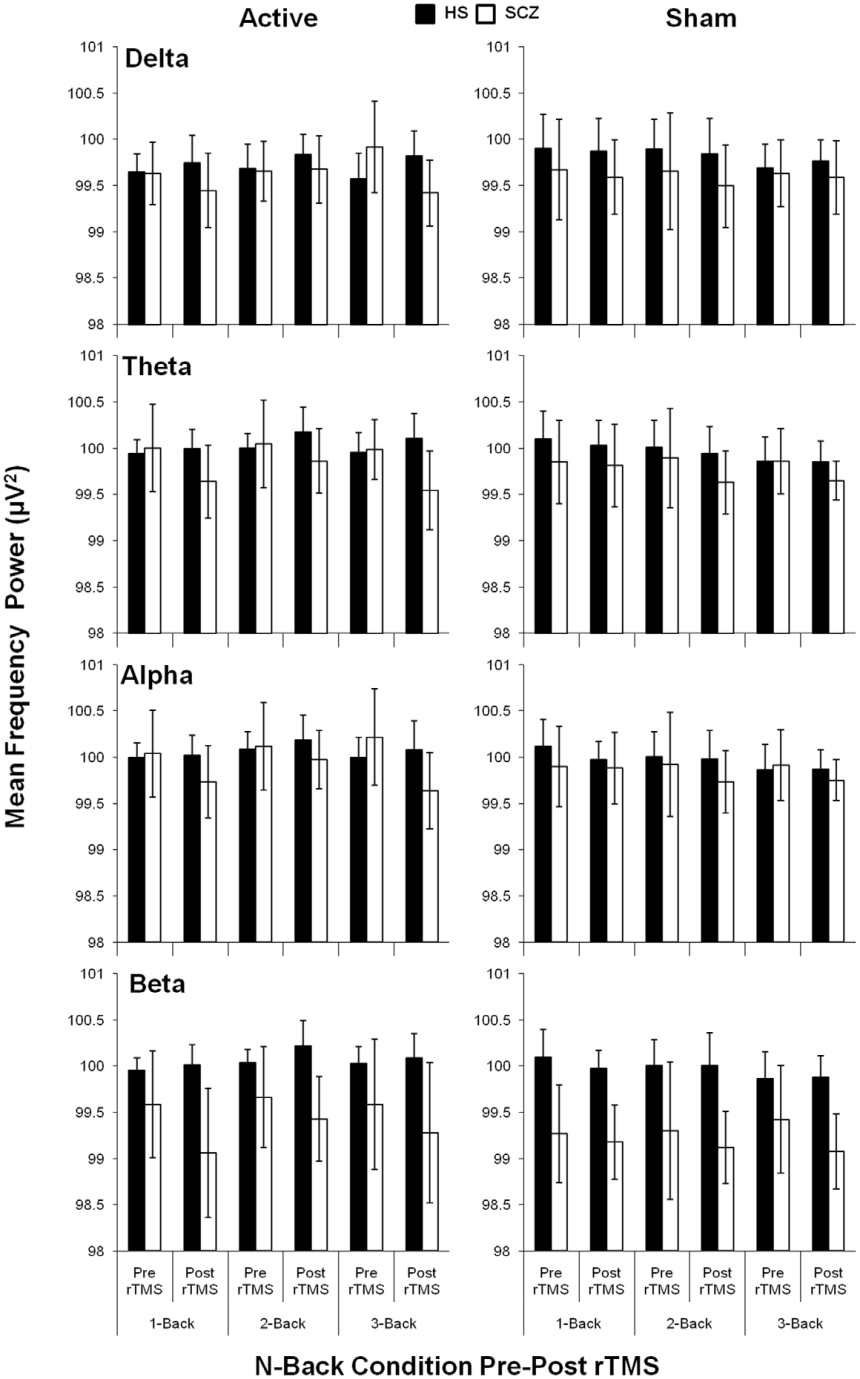
Mean log transformed oscillatory power (uV^2^) for target correct (TC) and non-target correct (NTC) responses during the N-back task pre-post rTMS in healthy subjects (HS; N = 22) versus patients with schizophrenia (SCZ; N = 24) across delta (δ; 1–3.5 Hz), theta (θ; 4–7 Hz), alpha (α; 8–12 Hz), and beta (β; 12.5–28 Hz) frequency ranges. Bars represent (±) 1 standard deviation.

Although γ oscillatory activity is most closely associated with higher order cognitive tasks, we explored the effect of rTMS on the mean change in oscillatory activities (post rTMS power-pre rTMS power) in the other frequency bands (δ, θ, α, and β) with four separate MMRM analyses. Although we observed a significant effect of Group on the change in mean oscillatory power in the θ, α, and β frequency bands, no significant Group×rTMS interactions were observed (Figure 4). However, there was a significant Group×rTMS×N-back interaction found in the δ frequency band such that active rTMS reduced activity in patients with SCZ in the 3-back condition compared to sham stimulation (p = 0.0048; Figure 4). To examine if the reduction in δ power was related to the reduction in γ power, a Pearson correlation coefficient was conducted and found significant positive relationships in the 1-back (r = 0.745; p = 0.008) and 3-back (r = 0.779; p = 0.008). No differences were found in healthy subjects in δ activation following rTMS administration.

### Effect of Antipsychotic Medication

A Pearson correlation coefficient was performed to determine if the changes in γ and δ oscillatory activity in the 3-back were related to antipsychotic medication using chloropromazine equivalents (CPZ; [41]) in the SCZ patient group. No relationships were found between γ or δ oscillatory activities and CPZ equivalents in the 3-back pre or post rTMS administration.

## Discussion

Consistent with our previous report [3], patients with SCZ elicited excessive frontal γ and reduced frontal β oscillatory activity compared to healthy subjects prior to rTMS. Following rTMS, excessive frontal γ oscillatory activity in SCZ patients was significantly reduced following bilateral rTMS to DLPFC. By contrast, rTMS significantly potentiated γ oscillatory activity in healthy subjects. These effects were most pronounced in the 3-back and were specific to the frontal cortical regions. rTMS also reduced δ activity in patients only. These results suggest that rTMS to DLPFC reduces excessive frontal γ oscillatory activity during the N-back in SCZ patients an effect that was opposite to that observed in healthy subjects.

The opposing effect of rTMS on γ oscillatory activity in patients and healthy subjects may be related to differential changes in GABAergic activity. For example, Daskalakis et al. (2006) demonstrated that 20 Hz rTMS applied to the motor cortex in healthy subjects had different effects depending on level of baseline GABAergic inhibitory neurotransmission. That is, rTMS potentiated short interval cortical inhibition (SICI), a neurophysiological paradigm that is related to GABA_A_ receptor inhibition [42], in subjects with relatively low baseline SICI and suppressed SICI in subjects with relatively high baseline activity [21] suggesting that rTMS can produce variable effects on GABA_A_ receptor mediated inhibition depending on baseline levels. As GABA_A_ inhibitory post synaptic potentials are involved in generation of γ oscillations [12], [13], [14] such findings can be used to explain the variable effects of rTMS on γ oscillatory activity in SCZ patients and healthy subjects. That is, rTMS inhibited γ oscillatory activity in SCZ patients with relatively greater γ activity at baseline, while potentiating activity in healthy subjects with relatively lower γ activity at baseline. Such effects may also be related to homeostatic plasticity, a brain mechanism that maintains neuronal activity within a useful physiological range and is critical to neuronal stability [43]. There is considerable evidence for the role of GABA_A_ receptor activity in the regulation of homeostatic plasticity [44], [45], [46], [47], [48] by modulating the number of post-synaptic GABA_A_ receptors that are activated to increase or decrease inhibitory neurotransmission [46], [49], [50], [51]. Regulation of GABA_A_ receptors in homeostatic plasticity have also been shown to be involved in the synchronization of neuronal activity [52], [53], [54], [55]. The opposing effect of rTMS on γ oscillatory activity in the current study, therefore, may reflect differential regulation of inhibitory activity through efficacy of GABA_A_ receptors important in homeostatic plasticity and generation of γ oscillations.

Alternatively, the effect of rTMS on γ oscillatory activity may reflect the regulation of cortical excitability to maintain homeostatic plasticity as GABA neurons are dependent on excitatory drive in generation of γ oscillations [12], [14], [56], [57], [58]. In this regard, the main source of neuronal excitation is through release of glutamate which typically activates N-methyl-D-asparate (NMDA) and non-NMDA receptors in the post-synaptic membrane [59]. The duration of non-NMDA excitatory post synaptic potentials (EPSPs) are optimal for fast signaling and coincidence detection. As such, non-NMDA EPSPs are important in the precise control of spike timing needed in the synchronization of cortical oscillations. The generation of oscillatory activity, therefore, may not only depend on GABA mediated inhibition but also on the recruitment of interneuron firing by glutamate excitation [59]. Homeostatic plasticity has been shown through the alteration of cortical excitability with transcranial direct current stimulation (tDCS) and rTMS administered to the motor cortex in healthy subjects [60]. Siebner et al. (2004) demonstrated that, cathodal tDCS reduced corticospinal excitability followed by 1 Hz rTMS that resulted in a sustained increase in corticospinal excitability. By contrast, increased corticospinal excitability by anodal tDCS was subsequently reduced with 1 Hz rTMS. Those subjects with the greatest changes induced by tDCS priming also exhibited the greatest change in corticospinal excitability following rTMS [60]. Siebner et al. (2004) therefore demonstrate that rTMS can produce variable effects on cortical excitability depending on baseline activity level. Given the importance of excitatory drive on γ oscillations, the homeostatic regulation of cortical excitability through rTMS may also have produced our finding of opposing effects on γ oscillatory in patients versus healthy subjects.

As previously shown, rTMS had no effect on the δ, θ, α, and β frequency bands in healthy subjects [3], that included an overlap of 82% of the subjects tested in the current study. In SCZ patients, however, rTMS reduced δ oscillatory activity in the 3-back compared to sham stimulation. This finding is consistent with a previous rTMS study in patients with SCZ with predominant negative symptoms [61]. For example, Jandl et al. (2005), reported a reduction in negative symptoms and δ oscillatory activity when rTMS was applied at 10 Hz to the left DLPFC for 5 days a [61]. Furthermore, it is possible that the reduction of δ activity following rTMS in SCZ patients observed in the current study was related to reduction of γ oscillatory activity reflecting the non-random relationships between oscillatory frequencies [62]. That is, cross-frequency interactions or “nesting” is observed when the power of a discrete frequency band is modified by the phase of a lower frequency band that coexists during information processing [62]. In this regard, hierarchies of nested rhythms have been observed in the neocortex [63] between δ, θ, and γ oscillatory activities. These findings suggest that γ oscillations are nested within δ oscillations that may explain the concomitant reduction in both activities following rTMS.

The current study contributes to the considerable evidence associating abnormal prefrontal functioning with WM impairment in patients with SCZ. However, the nature of prefrontal dysfunction remains controversial. That is, previous studies have reported similar [34], reduced [6], [64] and increased [2], [5] γ oscillatory activity in patients with SCZ compared to healthy subjects while performing WM tasks. Similar divergent findings have also been reported in fMRI studies that may be accounted for the sensory domain of the WM tested. In this regard, Brahmbhatt et al. (2006) tested patients with SCZ and their first degree relatives and reported hyperactivity of the prefrontal cortex during verbal WM while hypoactivation was observed when this task was administered in the visual domain [65]. Alternatively, differences in the activation of the prefrontal cortex may result from differences in the performance levels of patients with SCZ. For example, Callicott et al. (2003) observed both hyper- and hypoactivation of the prefrontal cortex during the N-back task in patients with SCZ compared to healthy subjects [66]. However, when patients were compared in terms of performance, it was found that low performers generated low activity while high performers generated relatively higher activity. Furthermore, when high performing patients were compared to high performing healthy subjects, hyperactivation of the prefrontal cortex was observed. Callicott et al. (2003) therefore demonstrate that at equivalent performance levels, patients with SCZ show hyperactivation of the prefrontal cortex that is consistent with our previous study [5] and prior to rTMS in the current study. The nature of prefrontal abnormalities underlying WM impairments continues to be debated. Moreover, factors such as performance levels, sensory domain, experimental paradigms, antipsychotic medication and the heterogeneity in the DLPFC warrants further examination vis à vis measuring γ oscillatory activity during WM in patients with SCZ.

### Limitations

This study is limited in some important ways. First, although rTMS decreased excessive γ oscillatory activity in SCZ patients and potentiated activity in healthy subjects, this change was not related to improved WM performance and may suggest that the relationship between γ oscillatory and WM performance may be epiphenomenal. Previous studies, however, have shown that rTMS induced cognitive changes are either delayed or optimal at some later time point [67], [68] and that repeated rTMS sessions may be needed to produce changes in gene expression and synapse formation associated with changes in short-term plasticity and cognition [69]. Nevertheless, this study provides early and interesting neurophysiological evidence for the modulating effect of rTMS on γ oscillatory activity, a finding that warrants further investigation as a potential therapeutic mechanism which underlies WM impairments in SCZ. Second, the relatively small sample size tested may be insufficient to detect an improvement in performance on the N-back following rTMS. Replication studies may consider using a larger sample size to examine this relationship. However, the fact that rTMS altered γ oscillatory activity compared to sham, suggests that the change in γ was not related to the small sample size. Nevertheless, such findings should be replicated in a larger sample to minimize Type II error and stabilize statistical parameter estimates [70]. Third, our finding of reduced γ oscillatory activity following rTMS may be related to the effect of anti-psychotic medication. In this regard, Hong et al. (2004) reported enhanced 40 Hz oscillations in SCZ patients that were treated with second generation compared those taking conventional antipsychotics [71]. In this study, oscillations were parsed into 20, 30 and 40 Hz; however, 30–50 Hz range is most conventionally examined during cognitive tasks [72]. Similarly, a differential effect of antipsychotic medication on cognitive performance has also been reported with SCZ patients on second generation performing better than those patients on conventional antipsychotics on a variety of cognitive tests, including WM [73], [74], [75]. In our sample, only 4 subjects were on conventional antipsychotics and these subjects happened to be randomly assigned to the sham group. Nevertheless, there were no differences found in γ oscillatory activity or in WM performance pre or post sham rTMS in those patients on conventional versus second generation antipsychotics. We were unable therefore to evaluate the effect of rTMS on γ oscillatory activity in SCZ patients on conventional versus second generation antipsychotics. This study did not include measures of neurotransmitter activity or dynamics pre or post rTMS and represents a final limitation to this study. For example, indices of GABAergic activity have been shown to be measured reliably through combined TMS-EEG techniques from both motor and the DLPFC in healthy subjects and patients with bipolar and SCZ [76], [77], [78] that may provide a greater understanding of the mechanism through which rTMS exerted the effects observed in this study. In addition, combined rTMS-PET can be used to detect changes in the level of extracellular dopamine [79] which could provide a better understanding of the action of rTMS on oscillatory activity. Future studies measuring indices of neurotransmitter action and dynamics through TMS-EEG or PET pre- and post-rTMS could provide a better understanding of how rTMS exerts its action on oscillatory activity in patients with SCZ.

### Summary

In summary, we demonstrated that rTMS over DLPFC alters frontal γ oscillatory activity, with the greatest effect at higher WM loads. In patients, rTMS reduced excessive γ oscillatory activity across WM load. In contrast, rTMS potentiated γ oscillatory activity in healthy subjects. The differential effect of rTMS on γ oscillatory activity may be related to the concept of homeostatic plasticity involving the regulation of GABAergic inhibitory mechanisms that maintain neuronal excitability within a useful physiological range. These findings provide important insights into the neurophysiological mechanisms that may lead to cognitive potentiation in this disorder.
